# Comprehensive Analysis of Machine Learning Models for Five-Class Sleep Stage Classification Using PPG Signals

**DOI:** 10.21203/rs.3.rs-7713914/v1

**Published:** 2026-08-10

**Authors:** Rafael Martins, Rita Ribeiro, Hugo Pereira, Vasco Silva, Alberto Freitas, Rute Almeida, Goreti Marreiros, Luís Conceição

**Affiliations:** 1 GECAD, Institute of Engineering, Polytechnic of Porto, Porto, Portugal; 2 RISE-Health, Department of Community Medicine, Information and Health, Decision Sciences, Faculty of Medicine, University of Porto, Porto, Portugal; 3 LASI, School of Engineering, University of Minho, Guimarães, Portugal

**Keywords:** Sleep Staging, Sleep Monitoring, Machine Learning, Photoplethysmography, Inter-Beat Interval, Wearable Monitoring

## Abstract

Accurate classification of sleep stages is essential for diagnosing sleep disorders and understanding sleep quality. While polysomnography (PSG) remains the gold standard, its invasiveness and cost hinder large-scale deployment. Photoplethysmography (PPG) offers a non-invasive and wearable alternative, yet most prior work either simplifies staging into fewer than five classes or relies on deep learning approaches with high computational demands. This study systematically evaluates traditional machine learning models for five-class sleep stage classification using PPG data from the MESA Sleep Dataset. A subset of 48 participants was analyzed, with signals segmented into 3-minute windows to enable robust extraction of both PPG-derived and inter-beat interval (IBI) features. Models including Random Forest, k-Nearest Neighbors, and XGBoost were benchmarked, demonstrating that combining PPG and IBI features substantially improves performance. Random Forest achieved the best results, with an accuracy of 72.2% and Cohen’s Kappa of 0.65, comparable to state-of-the-art deep learning methods while requiring fewer resources and offering greater interpretability. These findings highlight the potential of feature-based, computationally efficient models for five-stage sleep classification, supporting scalable and clinically relevant applications of wearable PPG monitoring.

## Introduction

Sleep plays a fundamental role in maintaining overall health and well-being, influencing critical cognitive, immune, and metabolic functions. Adequate sleep is essential for physical restoration, memory consolidation, and emotional regulation. However, disruptions in sleep, such as insomnia, sleep apnea, and other sleep-related disorders, are strongly associated with a wide range of chronic health conditions, including cardiovascular diseases, diabetes, obesity, hypertension, depression, and anxiety [1–4].

Sleep is organized into a sequence of physiologically distinct stages: light sleep (N1), intermediate sleep (N2), deep slow-wave sleep (N3) and rapid-eye-movement (REM). This sequence recurs in cycles of roughly 90–110 minutes each night [5]. The relative frequency and cumulative duration of these stages are widely used indicators of sleep quality, with imbalances linked to cardiovascular, metabolic and mental-health disorders [6–8]. Given these profound health implications, accurate and timely monitoring of sleep stages is essential for effective diagnosis and management of sleep-related conditions, ultimately improving quality of life.

Traditionally, polysomnography (PSG) has been the gold standard for studying sleep stages. PSG employs a comprehensive multimodal approach to monitor physiological signals such as the electroencephalogram (EEG), electrooculogram (EOG), electromyogram (EMG), and electrocardiogram (ECG), providing a detailed view of sleep stages, often defined using guidelines such as the AASM rules or the Rechtschaffen and Kales (R&K) criteria [9,10]. However, despite its diagnostic accuracy [11,12], PSG remains costly [12–14], invasive, intrusive and uncomfortable during sleep (potential disruption of natural sleep patterns of patients) [12,14,15], and typically requires patients to be monitored in clinical or laboratory environments [11]. While at-home polysomnography has been reported, it still shows substantial limitations in diagnostic accuracy compared to traditional in-lab PSG [11]. For these reasons, its practicality for large-scale and long-term use is restricted [16].

Recent advancements in wearable monitoring technologies offer a promising alternative to traditional PSG. While only the use of ECG is also a viable alternative, it remains more intrusive compared to PPG. In contrast, photoplethysmography (PPG), a non-invasive technique that measures blood volume changes through light absorption, has gained significant attention in this domain. Depending on the body site, PPG sensors can be transmissive, used for distal areas like fingers, or reflective, applied to locations like the forehead or wrist [17]. PPG signals, commonly obtained from wearable devices such as smartwatches, provide valuable information about heart rate (HR) and its variability (HRV), which can be used to infer sleep stages indirectly. Compared to PSG, PPG is less intrusive, more cost-effective, and more easily integrated into daily life, making it an appealing option for continuous sleep monitoring in clinical and home settings [13,18].

Although several studies already report strong performance for PPG-based sleep-stage classification (particularly when the task is limited to two, three or four classes [19]), this line of research is far from settled. PPG does not measure brain activity directly, as EEG does, it infers sleep stages indirectly through heart-rate-derived signals such as HR and HRV [20]. Extracting stage-specific information from these proxies demands sophisticated feature-engineering methods (like time-frequency analysis [21] or wavelet transforms [22] to capture relevant information) and raises questions about model robustness across populations and recording conditions [23]. Nevertheless, distinguishing all five AASM stages is clinically valuable, because the widely adopted four-class approach merges N1 + N2 into a single “light-sleep” category, thereby losing physiologically distinct information about arousal thresholds and autonomic tone [24,25].

To address these open issues, we systematically evaluate a range of traditional machine-learning models on a full five-stage problem, using feature sets designed to capture both time- and frequency-domain characteristics of the PPG signal. This approach allows us to quantify how much performance is gained (or lost) when moving to the more granular and clinically informative five-class framework while relying only on a single, wearable sensor modality.

This study aims to bridge these gaps by evaluating the performance of PPG-based 5-class sleep stage classification models, addressing the complexities of feature extraction and preprocessing. We employ traditional models and compare their performance with both traditional and neural network-based approaches reported in the literature.

## Related Work

Wearable photoplethysmography offers a non-invasive, cost-effective way to characterize sleep patterns and identify possible abnormalities, providing added value when conventional polysomnography cannot be deployed. Recent advancements in machine learning and deep learning techniques have significantly improved the performance of sleep staging models, with several studies demonstrating their potential using PPG-derived signals. But one thing that differentiates models is the number of sleep stages predicted, since higher granularity is harder to differentiate in sleep. The standard sleep classification has 5 stages: wake, N1, N2, N3, and REM; or 3 stages: wake, non-REM and REM. While various models have success when consider different number of classes (e.g. 2 classes: wake or sleep; or 3 classes: wake, non-REM or REM), predicting 4 (wake, “light-sleep N1+N2”, N3 or REM) or 5 (wake, N1, N2, N3, and REM) number of classes in sleep is harder.

Among the reviewed studies for 5-class sleep stage classification, as presented in Table 1, Braga et al. [26] employed a Multilayer Perceptron (MLP) achieving the highest accuracy of 80.5% and a Cohen’s kappa of 0.75.

Korkalainen et al. [27] implemented a convolutional neural network (CNN) combined with a recurrent neural network (RNN), achieving an accuracy of 64.1% (k = 0.51), highlighting the benefits of combining spatial and temporal features. Similarly, Kazemi et al. [28] utilized a CNN combined with a biderectional LSTM (BiLSTM), obtaining 77% accuracy (k = 0.67), showcasing the effectiveness of LSTM networks in capturing temporal dependencies in PPG signals.

Hybrid architectures have also shown promise. Huttunen et al. [29] combined CNN, LSTM and densely connected layers, reaching 68.7% accuracy (κ = 0.60), underscoring the value of mixed deep-learning pipelines. By contrast, several feature-based ensemble methods perform as well or better: Zhao et al. [19] reported 72% accuracy with LightGBM (κ = 0.64), and Braga et al. [26] achieved 76.4% with a Random Forest. These results indicate that well-engineered classical learners can rival deeper networks while demanding far less computation and offering greater interpretability, advantages that are critical for battery-powered wearables and for clinical acceptance.

Motivated by this gap, our study benchmarks a suite of traditional machine-learning models on the more challenging 5-stage problem to quantify how much performance can be recovered without resorting to heavyweight deep architectures.

All the studies reviewed utilized a consistent 30-second interval for data segmentation, ensuring comparability across results. The table below summarizes these key studies, their proposed models, intervals, and performance metrics:

These studies illustrate the growing role of both traditional machine learning and advanced deep learning techniques in PPG-based sleep stage classification. While models such as Random Forest, MLPs and LightGBM demonstrate strong performance with relatively low computational requirements, deep learning architectures (including CNNs, RNNs, and LSTMs) excel at capturing both local waveform morphology and long-range temporal dynamics in PPG signals. Hybrid approaches further highlight the potential for combining multiple methods to improve performance metrics.

Further research can focus on refining these models, exploring longer time intervals, and leveraging larger datasets to enhance generalizability and robustness across diverse populations.

## Materials

The RM4Health project focuses on the development of innovative frameworks for health monitoring and analysis, with particular emphasis on physiological signals such as photoplethysmography (PPG). As the project is still in its early development phase, no proprietary or real-world clinical data is currently available. Thus, for the initial validation of methodologies and models, publicly available datasets have been employed.

In this study, we utilize the MESA Sleep Dataset, which is part of the Multi-Ethnic Study of Atherosclerosis (MESA)[19]. This large-scale dataset investigates the relationship between sleep disorders and cardiovascular health in a demographically diverse cohort of over 2,000 participants. It includes a variety of data modalities such as polysomnography (PSG), actigraphy, and validated sleep questionnaires. PSG recordings encompass multiple physiological signals, including electrocardiography (ECG), electrooculography (EOG), electromyography (EMG), PPG, respiratory signals, and oximetry. Actigraphy provides sleep-wake metrics over a 7-day period, including Total Sleep Time (TST) and Wake After Sleep Onset (WASO).

For our specific purposes, we focused exclusively on PPG signals to assess their potential as a scalable and less invasive alternative to full PSG-based sleep staging. Access to the MESA Sleep Dataset was granted under strict data-sharing agreements, ensuring compliance with ethical standards and reproducibility requirements.

Each subject in the dataset is accompanied by detailed sleep stage annotations, available in XML files located in the /files/polysomnography/annotations-events-nsrr directory. PPG signals were extracted from the corresponding **.edf** sensor files in **the /files/polysomnography/edfs directory**. Sleep stage labels follow the AASM (American Academy of Sleep Medicine) standard, which defines five stages: Wake (W), Stage 1 (S1), Stage 2 (S2), Stage 3 (S3), and Rapid Eye Movement (REM) sleep.

Given the substantial size and heterogeneity of the full dataset, a representative subset of 48 participants was randomly selected in order to reduce the computational burden and manage the large data volume. Although modern systems can process large-scale datasets, the available computational resources in our setup were limited. This reduction allowed for a more feasible and efficient analysis during this initial phase of the project.

From each participant, 3-minute PPG segments were extracted for classification purposes. While many prior studies use 30-second epochs in alignment with standard PSG scoring, the 3-minute window was chosen here to capture more temporal context and potentially enhance the discriminative power of the features extracted, particularly when using models that leverage temporal dynamics. This segmentation strategy may also facilitate downstream comparisons with approaches based on longer temporal dependencies.

The segmentation and preprocessing procedures, including signal normalization and noise filtering, were performed by the authors d using custom Python scripts developed for this project. In total, 12,230 segments were extracted and retained for analysis before applying any data splitting or class balancing procedures.

The final distribution of the dataset across sleep stages is summarized below:

- Stage 2 (S2): 5,965 segments
- Wake (W): 3,057 segments
- REM: 1,361 segments
- Stage 3 (S3): 1,236 segments
- Stage 1 (S1): 611 segments

These annotations correspond to the official AASM-based scoring provided in the dataset.

## Methodology

This section describes the methodology utilized to read, process and organize the MESA dataset data as well as to train and test using it to train different traditional machine learning models. An overview of the steps taken is presented in the following diagram (Figure 1), with each step being further described in its respective subsection.

### Dataset Reading

As mentioned in the Materials section, this study uses a subset of 48 participants from the MESA dataset to train models capable of classifying sleep stages. In the MESA dataset, each PPG signal is bundled in a European Data Format (EDF) file named “mesa-sleep-<id>.edf”, while its respective sleep stage annotations are stored separately in XML files named “mesa-sleep-<id>-nsrr.xml”.

The objective of this step is to read both files and join each PPG signal (sampled at 256 Hz) with its respective sleep stages (measured every 30 seconds). To that end, first the PPG signal is extracted from the EDF file and resampled to 64 Hz, a frequency chosen as it is sufficient to preserve the physiological information of interest in PPG signals while reducing computational cost and storage requirements. Then, the signal is split into 30 second intervals, the sleep annotations are read from the XML file, and the two are joined by timestamp.

Each PPG interval was now labeled with its respective sleep stage, so this data could be used directly to train classification models, akin to the work of some existing studies (see Table 1). However, various articles advocate that many features extracted from PPG signals or from the derived Inter-beat Intervals (IBIs) require a signal length of at least 2 to 5 minutes [30–32]. These include PPG morphological features such as rise and decay times, and IBI-extracted features such as pNN50, RMMSD, VLH, LF and HF.

As such, an additional step was added to the data reading process, where the 30-second PPG signal intervals are grouped into longer duration segments. Grouping PPG intervals into longer segments does introduce a trade-off in temporal granularity, but it also enables more reliable feature extraction and noise mitigation. In particular, heart-rate-variability metrics like pNN50 and RMSSD, as well as low-frequency morphological characteristics, require at least 2–5 minutes of data to yield stable estimates [30,33]. Short 30-second epochs simply cannot capture these slower dynamics or consistently filter out motion artifacts. By using 3-minute segments, we ensure that each window contains enough information for robust calculation of both IBI-derived and waveform-based features, while cleaning out corrupted portions more effectively.

To preserve some temporal resolution, we employ a 1-minute sliding step: every six consecutive 30-second intervals are concatenated, assigned a single sleep-stage label via majority vote, and then shifted forward by two intervals (1 min) for the next segment (Figure 2). Although this scheme differs from the 30-second epochs used in prior five-stage studies, it allows us to explore whether richer, longer-window feature sets can match or exceed published 5-stage accuracies, potentially offering more robust performance in continuous, real-world monitoring scenarios.

As a result of this reading and grouping process, a dataset comprising 30196 labeled rows was obtained from the original 48 signal files.

### Pre-processing

Due to their nature, PPG signals are highly susceptible to noise and motion artifacts, which can significantly impact their signal quality.

Noise in PPG signals often arises from continuous or periodic sources, such as baseline drift or power line interference from the sensor [34]. Since this type of noise tends to affect only specific frequency bands, it can be effectively mitigated using frequency-domain filtering techniques. Motion artifacts, on the other hand, introduce large amplitude distortions that render sections of the signal uninterpretable. These artifacts, along with temporary signal flatlining caused by poor sensor placement or movement, result in segments where no meaningful physiological information can be extracted. Such sections must be identified and removed to ensure that only valid signals are used to train and evaluate the machine learning models. To address these challenges, this preprocessing stage consisted of the following two steps.

#### Signal Quality Assessment

Each 3-minute segment of the PPG signal was evaluated for quality using two criteria:

- The segment’s signal amplitude, defined as the difference between its maximum and minimum values, was required to fall within the range of 0.05–2.0. These thresholds were empirically defined based on exploratory analysis of the MESA dataset, ensuring that physiologically plausible segments were retained while discarding corrupted signals. While absolute amplitude ranges are not standardized, previous studies emphasize the importance of applying quality-control criteria and highlight that thresholds should be adapted to the dataset and application context [32,35].
- The number of detected peaks, which correspond to heartbeats, in each 3-minute segment must range between 90 and 330, corresponding to a heart rate range of 30 to 110 beats per minute (bpm). This peak quantity range was selected because it covers the expected heart rate range for typical physiological conditions during awake periods, approximately 60–100 bpm [34], while also encompassing the lower heart rates typically observed during sleep, as well as the higher heart rates that may occur during awake periods. This criterion helps to remove any flatlined or otherwise invalid segments.

Segments failing to meet either criterion were excluded from further analysis, so that only valid physiological signals were used for feature extraction and model training.

#### Frequency Filtering

To attenuate noise and isolate the relevant physiological components, a band-pass Butterworth filter is applied, removing frequencies outside the 0.5–5 Hz range, which corresponds to the normal frequency range of a PPG signal [36].

### Feature Extraction

On one hand, a PPG signal can be processed to extract features directly related to its morphology, as well as time-domain and frequency-domain characteristics. On the other hand, since PPG signals are by nature related to ECG, the peaks in a PPG waveform also correspond to heart beats, and as such can be used to calculate Inter-Beat Intervals (IBIs), enabling the extraction of features in common with ECG analysis. However, this correspondence assumes effective electromechanical coupling, which may be disrupted in cases such as arrhythmias, potentially leading to discrepancies between ECG and PPG signals. Still, for most healthy individuals and under normal physiological conditions, PPG provides a reliable and non-invasive alternative for heart rate and variability analysis, making it a practical tool for large-scale or continuous monitoring.

One advantage of IBI-based features is that, under conditions of adequate signal quality, they are less influenced by minor morphological noise present in raw PPG waveforms. However, IBIs remain highly sensitive to motion artifacts and peak detection errors, which can severely compromise their reliability [32,35,37]. In addition, since these features can also be derived from ECG, they provide a standardized feature extraction methodology, making it possible to switch between signal types for IBI calculation if necessary.

Conversely, direct PPG features, although more noise prone, may be able to capture unique and valuable information, such as blood pressure-related parameters, that are not reflected in IBIs alone. It is for this reason that this paper explores the separate and combined use of these two feature sets, to compare the strengths and results of these approaches. The Table 2 below summarize the features extracted from each raw PPG signal and the Table 3 summarize the features extracted from each PPG-derived IBIs.

Inter-Beat Intervals (IBIs) were calculated by detecting the peaks in the PPG waveform, which correspond to individual heartbeats. The time intervals between consecutive peaks were computed to obtain the IBIs, which serve as the basis for subsequent feature extraction.

### Data Preparation

With the features extracted in the previous stage and their corresponding sleep stage labels, machine learning models could theoretically be trained and evaluated. However, a two-step data preparation process was applied to address key challenges associated with traditional machine learning, specifically the bias introduced by unbalanced data and the potential for poor model convergence due to non-normalized features.

#### Data Balancing

Sleep stages are not evenly distributed throughout the night, resulting in an imbalance in the extracted 3-minute segments. To address the class imbalance in the dataset, a two-step balancing strategy was implemented for the training set. The goal was to set the target number of samples per class to 4473, which corresponds to the most represented class, namely Stage 2. Minority classes were oversampled using the Synthetic Minority Over-sampling Technique (SMOTE), which generates synthetic samples through interpolation in feature space. This approach resulted in a balanced training set with equal representation across all sleep stages.

For the test set, only random undersampling was used. All classes were reduced to match the size of the smallest class, which contained 153 samples. This ensured a balanced and unbiased evaluation set without the introduction of synthetic data.

#### Normalization

To facilitate model convergence, the features of each segment were individually normalized using min-max scaling. The normalization formula is:

$$X_{\text{norm}}=\frac{X-X_{\text{min}}}{X_{\text{max}}-X_{\text{min}}}$$

Here, *X* represents the original feature value, *X*_*min*_ is the minimum value of the feature, and *X*_*min*_ is its maximum value. This transformation scales all features to a range of [0, 1], which helps ensuring consistency across features and improves performance for models sensitive to feature scaling, such as SVMs and k-NN.

### Model Training and Testing

Multiple models are trained and compared to evaluate their performance in classifying sleep stages. For each model, grid search is employed to identify the optimal hyperparameters (Table 4), while cross-validation is applied to minimize overfitting and ensure that the evaluation is both robust and generalizable across different subsets of the data.

## Results and Discussion

The results in Table 5 show that the combination of PPG and IBI features consistently improves classification performance across all machine learning models tested. Among the models, Random Forest achieved the highest performance, with an accuracy of 72.16% and a Cohen’s Kappa of 0.652. KNN followed closely, attaining an accuracy of 71.63% and a Cohen’s Kappa of 0.645, while XGBoost reached an accuracy of 69.54% and a Cohen’s Kappa of 0.619. These results reinforce the effectiveness of tree-based and nearest-neighbor methods in capturing complex patterns in the data, especially when using combined feature sets.

In contrast, the Gaussian Naїve-Bayes model and the Support Vector Machine performed poorly, indicating their inability to model the non-linear relationships present in the data.

The combination of PPG and IBI features provided significant improvements over using either feature set alone, as evidenced by increased accuracy and Cohen’s Kappa in all models. While PPG features capture broader physiological trends, IBI features offer detailed insights into heart rate variability, enriching the feature space. This suggests that the complementary nature of these metrics is crucial for effective sleep stage classification.

The application of feature selection techniques, which resulted solely in the removal of the features *ppg_mean* and *ppg_rise_time*, also resulted in further improvements to the main models, as evidenced by the increased accuracy of Random Forest (from 71.24% to 72.16%) and KNN (from 66.80% to 71.63%), as demonstrated in Table 6.

The detailed metrics for the three best-performing models (Random Forest, KNN and XGBoost) reveal key strengths and challenges. Both Random Forest and XGBoost showed high precision and recall in stages S1 and S3, reflecting their robustness in identifying these states. However, stage S2 proved to be more challenging for all models, possibly due to its transient nature and overlap with other phases in terms of signal characteristics.

Random Forest showed the best overall results, with high F1 scores for stages S3 (0.82) and REM (0.82), as well as particularly high revocation for stage W (0.89). KNN, on the other hand, had a balanced performance in all stages, with REM (F1-Score of 0.78) and S3 (0.79) standing out. XGBoost, although with slightly lower accuracy, stood out with high F1-Scores for the S3 (0.81) and REM (0.81) stages, which demonstrates its robustness to noisy data and non-linear interactions. Tables 7, 8, and 9 present the confusion matrices for the Random Forest, k-Nearest Neighbors and XGBoost, respectively, and provide the precision, recall, and F1-score for each sleep stage.

The overall lower performance for S2 classification highlights a key limitation in the current approach. Another limitation lies in the use of 3-minute windows instead of the more conventional 30-second segments. Although this choice does not necessarily increase operational complexity, it diverges from standard practice. Nevertheless, it aligns with prior literature, which suggests that longer windows are more appropriate given the nature of the extracted features and their temporal dynamics.

This study makes several specific contributions to advance the field of PPG-based 5-class sleep stage classification. By addressing the challenges of feature extraction, model optimization, and preprocessing strategies, it seeks to enhance the utility of PPG as a viable tool for sleep analysis. The key contributions include:

- **Implementation and Evaluation of Diverse Traditional Models**: This study employs a variety of machine learning models, such as Classification Tree, Random Forest, Support Vector Machine and XGBoost, to classify 5-class sleep stages from PPG data, following a systematic and modular approach that facilitates reproducibility and future extensions.
- **Prediction of Five Sleep Stages**: While many studies focus on 2-, 3-, and 4-class classifications, this work predicts all 5 sleep stages (Wake, S1, S2, S3, REM), providing a more detailed perspective on sleep architecture.
- **Comparison Across Models and Literature**: Results are benchmarked against other machine learning and neural network approaches and existing studies to assess the effectiveness of PPG as a viable alternative to PSG.

Through these contributions, this study seeks to advance the field of PPG-based sleep monitoring by demonstrating the potential of traditional models for predicting sleep stages, investigating approaches to improve the accuracy and reliability of sleep stage classification, and providing deeper insights into sleep architecture through the prediction of five distinct sleep stages.

Future studies could explore advanced feature engineering, such as incorporating frequency-domain analysis or hybrid architectures combining deep learning with traditional models, to better capture the nuances of transitional stages. Additionally, exploring domain-specific regularization techniques and multi-modal data fusion could further enhance classification performance.

## Conclusions and future work

This study highlights the significant potential of PPG signals as a scalable and non-invasive alternative to PSG for sleep monitoring, addressing key limitations of traditional methods. Combining PPG features with IBI data demonstrated substantial improvements in the performance of machine learning models, with Random Forest achieving an accuracy of 72.16% and a Cohen’s Kappa of 0.652. When compared to traditional approaches in the literature, these results are competitive with those reported in the literature and were only surpassed by Braga et al. [26], whose Random Forest model reached 76.4% accuracy and a Cohen’s Kappa of 0.70.

These findings validate the efficacy of PPG-based technologies for classifying all five sleep stages – Wake, S1, S2, S3, and REM – providing a detailed understanding of sleep architecture, although S2 presents some classification challenges due to its physiological similarity to other stages.

The practical implications of this research are extensive, particularly in enabling non-invasive, home-based sleep monitoring and supporting large-scale population studies, even though the proposed method does not outperform the state of the art. Nonetheless, its simplicity, reproducibility, and reliance on traditional models make it highly suitable for real-world deployment and integration into wearable technologies. Wearable devices that integrate PPG technology offer an affordable and accessible solution for continuous sleep analysis, enhancing diagnostic capabilities and promoting personalized health management.

Future research should focus on optimizing preprocessing techniques and feature extraction to address challenges such as noise susceptibility and class imbalance. Moreover, integrating hybrid machine learning and deep learning architectures for fully automated feature extraction and classification could further improve accuracy and robustness. Expanding datasets to include more diverse populations and exploring real-time applications will be essential for advancing the field of wearable sleep monitoring.

## Figures and Tables

**Fig. 1 -:**
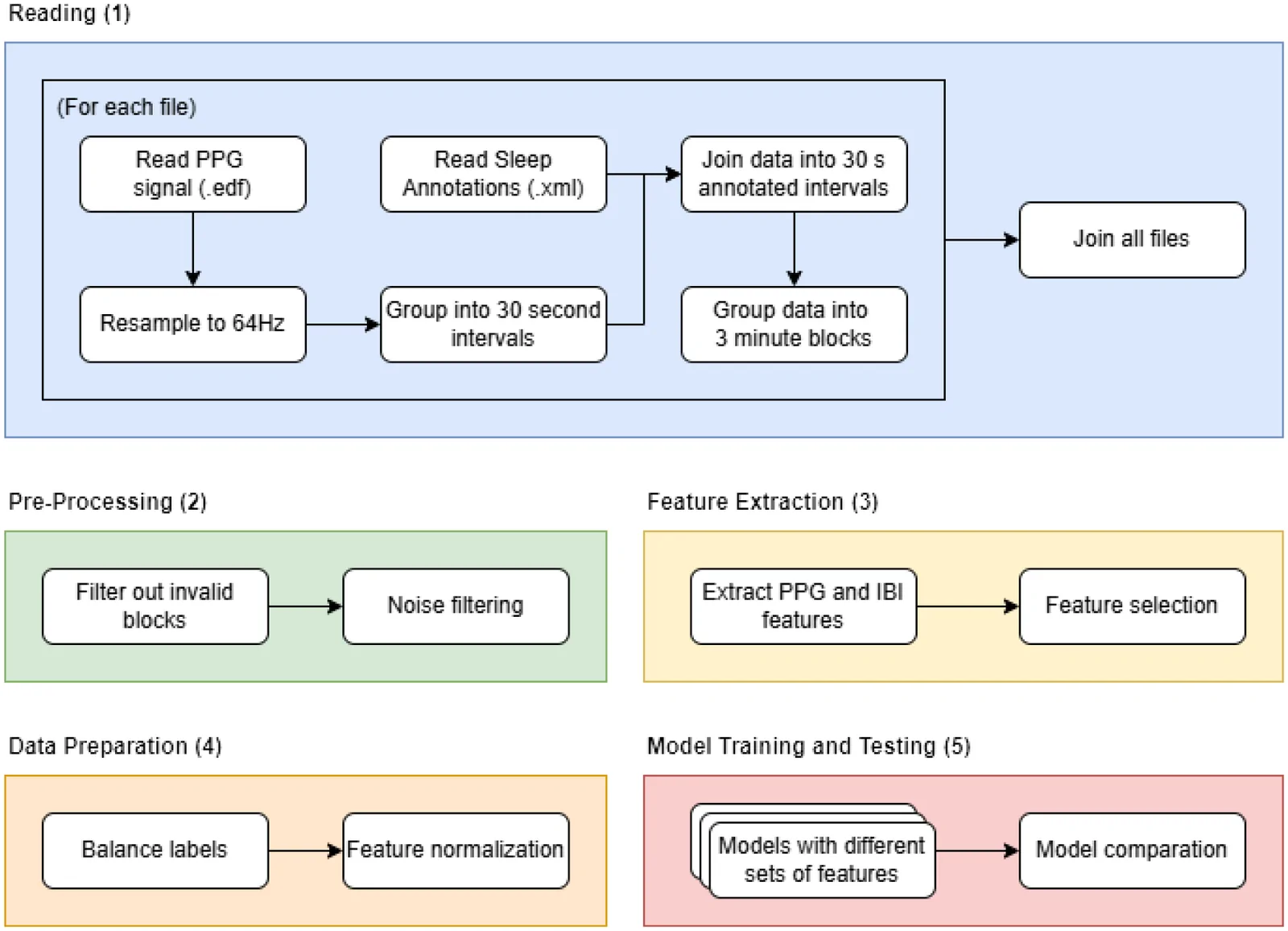
Methodology followed for data processing and model training

**Fig. 2 -:**
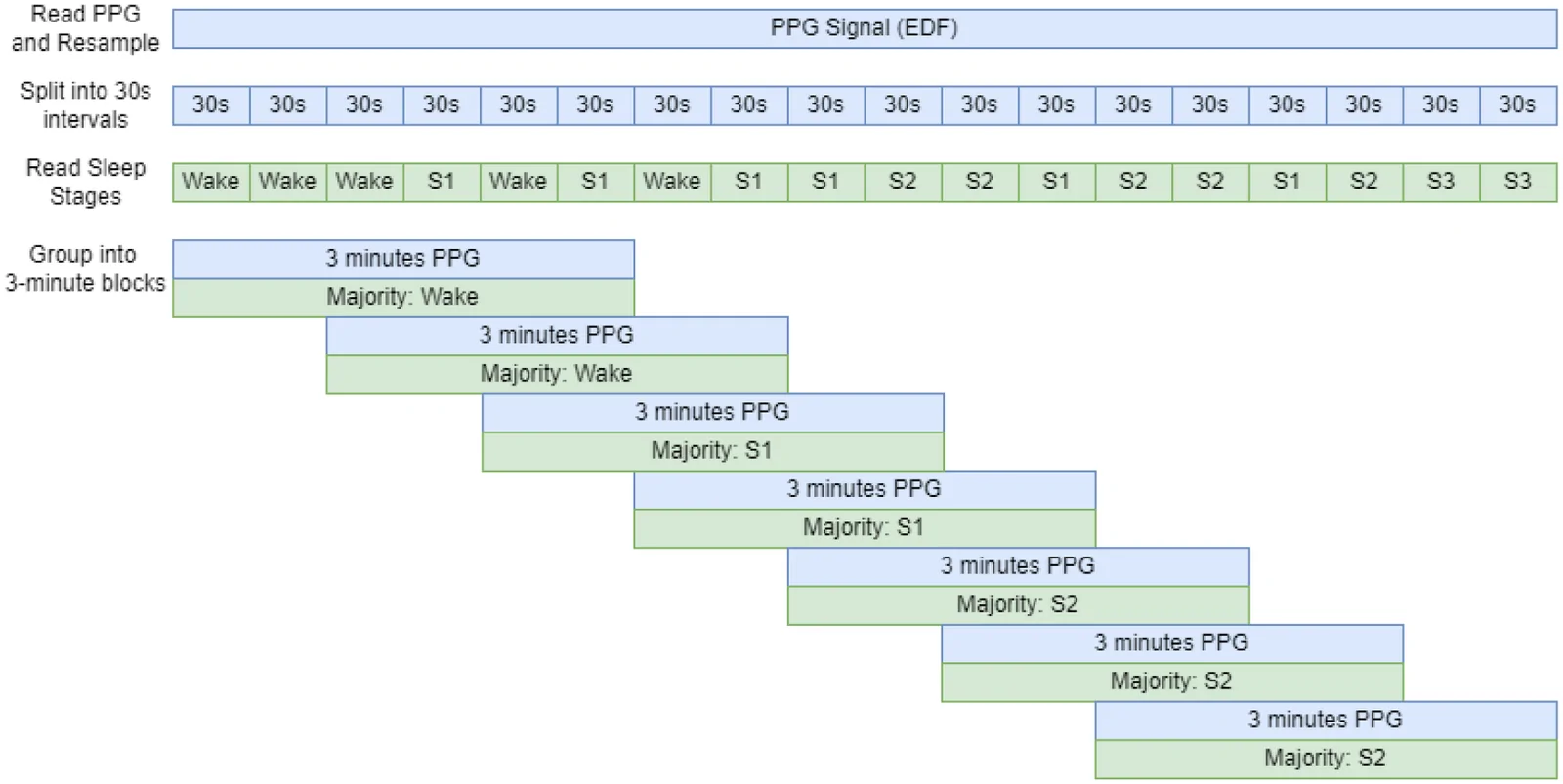
Data reading and grouping process

**Table 1 –:** Overview of prior studies on five-class sleep-stage classification: datasets, model types, epoch interval and key performance metrics.

Authors	Dataset	Sample Size (subjects)	Sleep stages predicted	Model	Interval	Accuracy (%)	Cohen-k
Braga et al. [26]	MESA	2056	5	Random Forest	30 sec	76.4	0.70
				Multilayer Perceptron	30 sec	80.5	0.75
Huttunen et al. [29]	Private	877	5	CNN + RNN (LSTM) + densely connected classifier	30 sec	68.7	0.60
Kazemi et al. [28]	Private	123	5	CNN + LSTM	30 sec	77	0.67
Korkalainen et al. [27]	Private	894	5	CNN + RNN	30 sec	64.1	0.51
Zhao et al. [19]	CAP Sleep Database	27	5	LightGBM (Light Gradient Boosting Machine)	30 sec	72	0.64

**Table 2 -:** Features extracted from each 3-minute segment PPG signal

Feature Type	Feature	Description
Descriptive Statistics	ppg_mean	Mean (average) value of the PPG signal
	ppg_median	Median of the PPG signal
	ppg_std	Standard deviation of the PPG signal
	ppg_mad	Median absolute deviation of the PPG signal
	ppg_iqr	Interquartile range (difference between 75th and 25th percentiles) of the PPG signal
Temporal	ppg_rise_time	Time it takes for the signal to rise above the median threshold
	ppg_decay_time	Time it takes for the signal to fall below the median threshold
	ppg_slope	Linear slope of the signal, indicating overall trend
Frequency	ppg_psd	Power spectral density, or signal energy (frequency range 0.04–0.4 Hz)
	ppg_peak_freq	Frequency of the peak power in the PPG signal spectrum
	ppg_cwt_energy	Total energy, calculated using continuous wavelet transform (CWT)
	ppg_fft_energy	Total energy, calculated using fast Fourier transform (FFT)
Heart Rate Related	ppg_bpm	Beats per minute, estimated from peak intervals
	ppg_peak_rate	Rate of detected peaks per unit time
Signal Shape	ppg_skewness	Skewness (asymmetry) of the signal distribution
	ppg_kurtosis	Kurtosis (“tailedness”) of the signal distribution
Signal Complexity	ppg _hjorth_activity	Signal variance, representing total energy (Hjorth method)
	ppg _hjorth_mobility	Estimate of the signal’s mean frequency, based on the first derivative (Hjorth method).
	ppg_hjorth_complexity	Degree of frequency variation over time, computed from the first and second derivatives of the signal (Hjorth method).

**Table 3 -:** Features extracted from each 3-minute segment IBIs

Feature Type	Feature	Description
Time Domain	ibis_std	Standard deviation of the IBIs
	ibis_mean	Mean (average) value of the IBIs
	ibis_rmssd	Root mean square of successive differences between IBIs
	ibis_pnn50	Percentage of successive IBIs differing by more than 50 ms
	ibis_nn50	Number of successive IBI pairs differing by more than 50 ms
	ibis_sdnn	Standard deviation of all IBIs (global HRV measure)
Frequency Domain	ibis_vlf_power	Power in the very low-frequency (VLF) band (0.0033–0.04 Hz) of the IBIs' power spectral density
	ibis_lf_power	Power in the low-frequency (LF) band (0.04–0.15 Hz) of the IBIs' power spectral density
	ibis_hf_power	Power in the high-frequency (HF) band (0.150.40 Hz) of the IBIs' power spectral density
	ibis_lfhf_ratio	Ratio of LF power to HF power
	ibis_spectral_entropy	Entropy of the normalized power spectrum, measuring frequency dispersion
	ibis_total_power	Total power in the VLF, LF, and HF bands
Non-Linear	ibis_sampen	Sample entropy (complexity / irregularity) of the IBIs
	ibis_sd1	Short-term variability (perpendicular spread in Poincare plot)
	ibis_sd2	Long-term variability (along-axis spread in Poincare plot)
	ibis_higuchi_fd	Fractal dimension via Higuchi method, representing signal complexity
	ibis_dfa	Detrended fluctuation analysis (long-term correlations) of the IBIs

**Table 4 -:** Models trained and respective hyperparameter variations

Model	Description	Hyperparameter	Values Tested
Classification Tree	Decision tree classifier	criterion	gini, entropy
		max_depth	None, 5, 10
		min_samples_split	2, 5, 10
Random Forest	Ensemble of decision trees	n_estimators	100, 150, 250
		max_depth	None, 5, 10
		min_samples_split	2, 5, 10
Gaussian NB	Naive Bayes with Gaussian assumption	var_smoothing	1e-9, 1e-8, 1e-7
KNN	k-Nearest Neighbors classifier	n_neighbors	3, 5, 10
		weights	uniform, distance
		metric	euclidean, manhattan
SVM	Support Vector Machine	C	0.1, 1, 10
		kernel	linear, rbf
		gamma	scale, auto
		degree	2, 3, 4
XGBoost	Gradient boosting decision trees	n_estimators	50, 100, 150
		learning_rate	0.01, 0.1, 0.2
		max_depth	3, 5, 7

**Table 5 -:** Initial results by model and by feature type

Model	IBI features only		PPG features only		IBI and PPG features	
	*Accuracy*	*κ*	*Accuracy*	*κ*	*Accuracy*	*κ*
Classification Tree	57.16	0.464	57.12	0.464	58.43	0.480
Random Forest	64.97	0.562	64.97	0.562	**71.24**	**0.641**
Gaussian NB	27.97	0.100	27.97	0.100	32.03	0.150
KNN	50.33	0.379	50.33	0.379	**66.80**	**0.585**
SVM	41.31	0.266	41.31	0.266	56.73	0.459
XGBoost	60.26	0.503	60.26	0.503	**67.19**	**0.590**

**Table 6 -:** Impact of feature selection on model performance

Model	IBI and PPG features		IBI and PPG features + Feature Selection	
	*Accuracy*	*κ*	*Accuracy*	*κ*
Classification Tree	58.43	0.480	57.91	0.474
Random Forest	71.24	0.641	**72.16**	**0.652**
Gaussian NB	32.03	0.150	32.16	0.152
KNN	66.80	0.585	**71.63**	**0.645**
SVM	56.73	0.459	58.69	0.484
XGBoost	67.19	0.590	**69.54**	**0.619**

**Table 7 -:** Confusion Matrix - Random Forest

Random Forest								
	W	S1	S2	S3	REM	Precision	Recall	F1-Score
**W**	136	5	7	3	2	0.69	0.89	0.77
**S1**	32	62	35	9	15	0.69	0.41	0.51
**S2**	14	14	107	7	11	0.59	0.70	0.64
**S3**	6	2	22	120	3	0.86	0.78	0.82
**REM**	10	7	9	0	127	0.80	0.83	0.82
Accuracy: 72.16%						Cohen’s Kappa: 0.652		

**Table 8 -:** Confusion Matrix – k-Nearest Neighbors

k-Nearest Neighbors								
	W	S1	S2	S3	REM	Precision	Recall	F1-Score
**W**	122	11	13	4	3	0.73	0.80	0.76
**S1**	20	81	26	7	19	0.69	0.53	0.60
**S2**	12	15	107	7	12	0.59	0.70	0.64
**S3**	4	4	25	117	3	0.82	0.76	0.79
**REM**	9	7	9	7	121	0.77	0.79	0.78
Accuracy: 71.63%						Cohen’s Kappa: 0.645		

**Table 9 -:** Confusion Matrix - XG Boost

XGBoost								
	W	S1	S2	S3	REM	Precision	Recall	F1-Score
**W**	132	4	13	3	1	0.70	0.86	0.77
**S1**	32	45	55	5	16	0.73	0.29	0.42
**S2**	11	8	118	7	9	0.51	0.77	0.61
**S3**	3	1	32	114	3	0.88	0.75	0.81
**REM**	11	4	15	0	123	0.81	0.80	0.81
Accuracy: 69.54%						Cohen’s Kappa: 0.619		

## Data Availability

This work is a secondary analysis of the MESA Sleep dataset hosted by the National Sleep Research Resource (NSRR). The data are publicly available from the repository upon free registration and acceptance of a data-use agreement (NSRR DOI: 10.25822/n7hq-c406). Data requests should be directed to the NSRR/MESA repository; the authors are not the data custodians. Further information about dataset access and NSRR policies is available on the repository website.
